# Pulse-resolved multi-photon X-ray detection at 31 MHz based on a quadrant avalanche photodiode

**DOI:** 10.1107/S1600577514006730

**Published:** 2014-06-03

**Authors:** Tobias Reusch, Markus Osterhoff, Johannes Agricola, Tim Salditt

**Affiliations:** a Institute for X-ray Physics, Georg-August University of Göttingen, Friedrich-Hund-Platz 1, 37077 Göttingen, Germany

**Keywords:** ultra-fast detection, time-resolved diffraction, multi-photon counting

## Abstract

The technical realisation and the commissioning experiments of a high-speed X-ray detector based on a quadrant avalanche silicon photodiode and high-speed digitizers are described. The development is driven by the need for X-ray detectors dedicated to time-resolved diffraction and imaging experiments, ideally requiring pulse-resolved data processing at the synchrotron bunch repetition rate. By a novel multi-photon detection scheme, the exact number of X-ray photons within each X-ray pulse can be recorded. Commissioning experiments at beamlines P08 and P10 of the storage ring PETRA III, at DESY, Hamburg, Germany, have been used to validate the pulse-wise multi-photon counting scheme at bunch frequencies ≥31 MHz, enabling pulse-by-pulse readout during the PETRA III 240-bunch mode with single-photon detection capability. An X-ray flux of ≥3.7 × 10^9^ photons s^−1^ can be detected while still resolving individual photons at low count rates.

## Abstract 2

The technical realisation as well as the first commissioning experiments of a high-speed X-ray detection scheme based on a quadrant avalanche silicon photodiode for single-pulse multiphoton detection of synchrotron radiation are described. It is shown that the detector is able to record the exact number of photons for each pulse continuously at a repetition rate of ≥31 MHz.

## 1. Introduction   

X-ray experiments at synchrotron or free-electron laser (FEL) sources are often limited by the dynamic range and readout time of current detector technologies rather than the peak brilliance of the X-ray source itself. An important example is time-resolved stroboscopic (pump–probe) diffraction experiments, where fast pulse selection or gating is required to obtain high temporal resolution in the picosecond range. Whether individual pulses are selected *via* electronic gating of single-photon-counting pixel detectors (Reusch *et al.*, 2013*b* ▶; Ejdrup *et al.*, 2009 ▶) or by high-speed mechanical choppers (Wulff *et al.*, 2003 ▶; Cammarata *et al.*, 2008 ▶), the usable flux is reduced by orders of magnitude.

**Figure 1:**
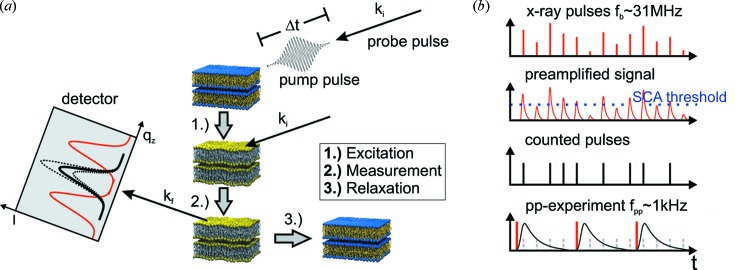
(*a*) Schematic of a laser pump/X-ray probe experiment on a multi-lamellar lipid stack, in the conventional set-up of low-frequency filling modes, by use of choppers/gating detectors. The temporal evolution of the specular and diffuse scattering is recorded to obtain information about the structural dynamics on a molecular scale in response to short pulse excitation. (*b*) Schematic of the operating principle of single-photon-counting detectors (row 1–3). A single-channel analyzer (SCA) discriminates events from background noise; individual events are counted in a binary fashion. Stroboscopic experiments at *f*_pp_ = 1 kHz (row 4) smaller than the synchrotron pulse frequency *f*_b_ ≃ 31 MHz (row 1) are necessarily accompanied by a tremendous loss in the effective X-ray intensity due to fast gating or mechanical pulse selection as indicated by grey shading.

Let us briefly consider the case of a time-resolved ‘optical pump–X-ray probe’ experiment such as sketched in Fig. 1(*a*) ▶, corresponding to a recent application in which we have studied the light-driven out-of-equilibrium dynamics in lipid multilamellar membranes (Reusch *et al.*, 2013*a* ▶). Ultrafast and fast dynamics are excited by a short (pulse length 50 fs ≤ τ ≤ 200 ns) laser pulse before the instantaneous sample structure is probed by a τ ≃ 50 ps X-ray pulse at a well defined time delay Δ*t*. This pump–probe scheme is repeated in a stroboscopic fashion at a frequency of typically *f*_pp_ ≃ 1 kHz for a fixed time delay Δ*t* until a suitable signal-to-noise ratio is achieved. The variation of Δ*t* then allows the structural evolution to be followed after short pulse excitation. The first generic observables in such a time-resolved scattering experiment are the position **q**(*n*, Δ*t*) and integrated intensity *II*[**q**(*n*, Δ*t*), Δ*t*] of individual reflections *n* as a function of delay Δ*t* (Reusch *et al.*, 2013*a* ▶,*b* ▶).

Current detector technologies do not allow for a pulse-wise recording of subsequent X-ray probe pulses at the native repetition rate *f*_b_ >> 1 MHz of synchrotron sources. Instead, temporally well defined (‘sharp’) snapshots at a given time delay Δ*t* are only obtained if the effective X-ray pulse frequency *f*_b_ is matched/reduced to the stroboscopic experiment at *f*_pp_ = 1 kHz. The temporal resolution is in this case, apart from jitter, given by the pulse length τ of the X-ray source; typical values range from τ ≃ 50 ps for synchrotron sources to τ ≤ 10 fs for FELs.

Single-pulse selection by means of high-speed mechanical choppers (Wulff *et al.*, 2003 ▶; Cammarata *et al.*, 2008 ▶) or gating of modern pixel array detectors (PADs) (Reusch *et al.*, 2013*b* ▶; Ejdrup *et al.*, 2009 ▶), for example at a frequency of *f*_pp_ = 1kHz, is thus necessarily accompanied by a tremendous decrease of effective X-ray flux. For a synchrotron or FEL source with high bunch frequency *f*_b_, this factor between available and used flux can become very significant; for example, *f*_b_/*f*_pp_ ≃ 31000 for the 240-bunch mode of PETRA III [*f*_b_ ≃ 31 MHz, see the sketch in Fig. 1(*b*) ▶]. This intensity loss poses significant restrictions on possible sample systems and leads to very long data accumulation times. Furthermore, individual X-ray pulse selection is often restricted to lower *f*_b_ than the optimum values for other experiments, limiting stroboscopic time-resolved X-ray experiments to specially scheduled low-frequency filling modes.

Beyond the advanced experimental requirements imposed by time-resolved scattering experiments, the binary nature of current single-photon-counting detectors imposes strict limitations on the dynamic range. Notably, the photon flux has to be limited to <<1 photon per pixel-pulse to avoid detector saturation. Furthermore, readout times in the millisecond range strongly limit the performance of diffraction and also of imaging experiments, for example in scanning transmission X-ray microscopy (STXM).

In conclusion, all present approaches to recording successive X-ray pulses at repetition rates of several MHz [*e.g.* the AGIPD (Henrich *et al.*, 2011 ▶), XNAP (Fajardo *et al.*, 2013 ▶) or CSPAD (Herrmann *et al.*, 2013 ▶) projects] are limited by either the maximum number of subsequently recordable X-ray pulses or the binary nature of single-photon-counting detectors. At the same time they offer the advantage of a large number of pixels, which are all treated in parallel. For the particular application of time-resolved reflectivity where one-dimension detectors are well suited to recording the strongly peaked specular signal, or for scanning transmission microscopy in phase-contrast mode of periodic processes (Van Waeyenberge *et al.*, 2006 ▶; Kammerer *et al.*, 2011 ▶), we present an improved detection mechanism in this work, which meets the following specifications:

(i) The detection must have single-photon sensitivity.

(ii) Multi-photon events (*N* ≤ 100 photons per pulse) must be resolved.

(iii) Changes in the direction of the scattered or transmitted beam should be recorded pulse-by-pulse.

(iv) Each event has to be time-stamped in order to enable sorting relative to a pump–probe experiment.

(v) Pulse-to-pulse online data analysis (*i.e.* position and correlation analysis, temporal binning, waveform averaging) is required to reduce the data stream.

(vi) The detector should be usable in high-frequency bunch modes, such as the 240-bunch mode of PETRA III (*f*_b_ ≃ 31 MHz). Analog as well as digital pulse processing thus has to be achieved in less than 30 ns.

## 2. Technical realisation   

In order to fulfil the above requirements, in particular (i), (ii) and (iii), the present approach is based on a quadrant avalanche photodiode (APD; QA4000, First-Sensor AG, Berlin, Germany) operating in the linear regime. We will refer to this detector as QAPD (quadrant avalanche photodiode). A silicon-based sensor is chosen because of its good commercial availability and the sufficient absorption properties for X-ray energies ≤20 keV (Henke, 1993 ▶). The use of APDs to detect X-rays, however, has been discussed in detail by Baron *et al.* (2006 ▶), but, in view of understanding the system presented here, we will include some basic explanation of the fundamental mechanisms and parameters in this work.

Let us first briefly estimate the current signal corresponding to the detection of an individual X-ray photon at an energy of 10 keV in a silicon diode. The ionization energy of undoped silicon is 3.6 eV (Knoll, 2010 ▶); one fully absorbed 10 keV X-ray photon will therefore create *N* ≃ 2800 electron–hole pairs. With an estimated width of τ = 20 ns of the electronic response of a fast silicon APD, this leads to an electronic current of *I*_p_ = 2800 e^−^/20 ns ≃ 22 nA. When operated in the linear regime, the avalanche effect of an APD provides a current amplification of 10–1000× (Hering *et al.*, 2005 ▶); a single-photon signal of *I*_p_ = 250 × 22 nA = 5.5 µA is therefore assumed as a starting point for the design of the subsequent pulse amplification and processing electronics.

**Figure 2:**
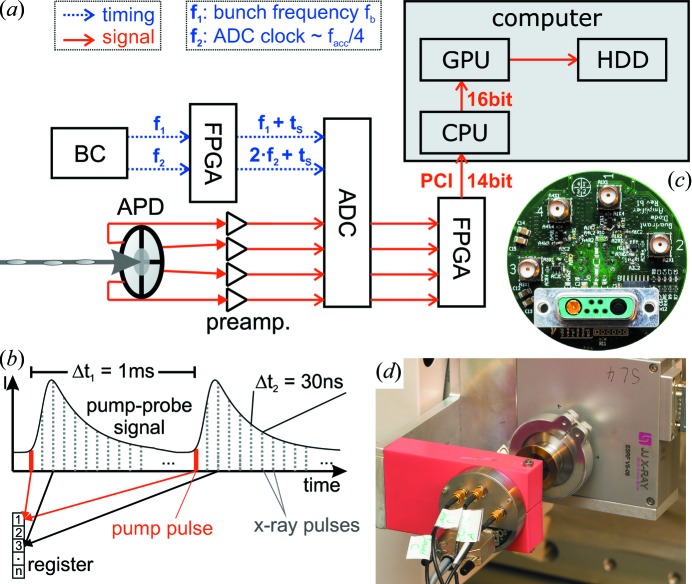
(*a*) Sketch of the data acquisition scheme. Individual X-ray pulses are detected by the APD; a fast preamplifier converts individual charge pulses into voltage signals. Readout by a high-speed analog-to-digital converter (ADC) is followed by FPGA-based data encoding and transfer through the PCI bus. Online data analysis can be performed by a graphics processing unit (GPU); the full data stream can be recorded. (*b*) Pulse-to-pulse online data analysis can be performed at bunch frequencies *f*_b_ >> 31 MHz. In the example of waveform averaging, individual X-ray pulses are stored in registers in the GPU depending on the time of arrival, the dynamics as induced at, for example, *f*_pp_ = 1 kHz (sketched in red) can therefore be probed in a stroboscopic manner. (*c*) Photograph (back side) of the high-bandwidth preamplifier PCB. The quadrant APD is located in the center; amplified signals are routed to four SMA connectors. Bias and supply voltages are provided *via* a mixed Sub-D connector. (*d*) Photograph of the detector head containing the quadrant APD as well as the preamplifier PCB as mounted at the beamline P08 at PETRA III.

The basic concept of the detection scheme is sketched in Fig. 2(*a*) ▶. Each channel of the quadrant APD is routed to a fast preamplifier circuit and digitized by a high-bandwidth analog-to-digital converter (ADC; FMC-104, 4DSP, USA). By synchronizing the sampling rate and phase to external trigger and clock sources provided by the synchrotron storage ring (Reusch *et al.*, 2013*b* ▶), the signal corresponding to individual X-ray pulses can be measured. Digital data are further analyzed and transferred to a personal computer (PC) by a field programmable gate array (FPGA; Virtex 6, Xilinx). Basic online data processing (integration, offset subtraction) can be performed in the FPGA prior to the data transfer, followed by more complex online data analysis in the graphics processing unit (GPU) of the PC. A more detailed description of the individual components will be given in the following paragraphs. For an in-depth explanation of the components, we also refer to the respective data sheets, as well as to Horowitz *et al.* (1989 ▶) for an advanced textbook level.

### 2.1. Detector front-end   

The detector front-end is a silicon-based quadrant APD with an active area of diameter 4 mm; individual elements are separated by a gap of 110 µm. The silicon layer has a thickness of 180 µm, leading to a detection efficiency of approximately 74% for 10 keV X-ray photons; higher detection efficiencies can be achieved for thicker depletion layers at the expense of slightly increased pulse widths (longer drift times). A linear gain (current amplification) of 250× is reached for a bias voltage of *U* ≃ 250 V. Note that the detector front-end can be easily exchanged. Smaller gaps or different materials can be introduced without interference with subsequent detection components.

#### 2.1.1. Preamplifier   

Current signals generated by the detector front-end are converted to voltage signals in an OPA-based low-noise–high-bandwidth trans-impedance amplifier (TIA). The electronic design of the custom-built TIA is based on the Texas Instruments LMH6629 OPA (specified −3 dB bandwidth ≤900 MHz). We refer to the product data sheet for the electronic circuits. Values for all electrical components have first been estimated according to the data sheet of the OPA and then been optimized in an iterative manner during three commissioning beam times. All commissioning experiments for the analog signal chain as presented in this section have been performed during the 240-bunch mode of PETRA III (*f*_b_ ≃ 31 MHz) at beamline P08; the photon energy was 18 keV. High-bandwidth oscilloscopes (Lecroy WaveRunner 640Zi, Tektronix DPO4104B, Tektronix DPO7254C) have been used to analyze the analog signal.

**Figure 3:**
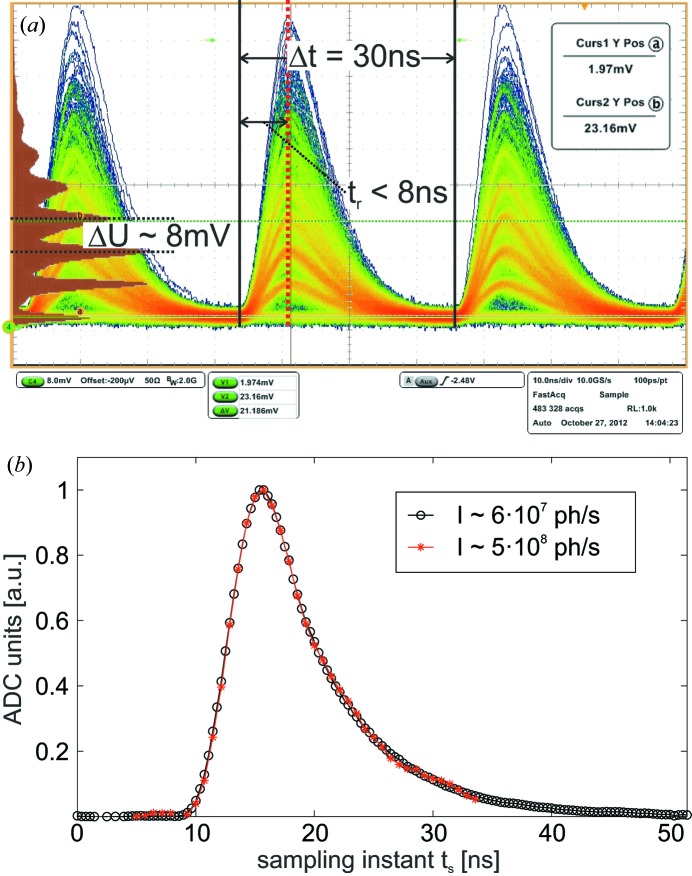
(*a*) Oscilloscope trace of the preamplified signal of an individual channel of the APD for a primary intensity of *I*_0_ ≃ 6 × 10^7^ photons s^−1^ (detected photons) along with a histogram of the peak pulse height (plotted in brown). Experiments have been performed at a photon energy of *E* = 18 keV during the PETRA III 240-bunch mode (*f*_b_ ≃ 31 MHz). Individual photon population numbers can clearly be distinguished in the pulse-height histogram. Pulse widths are τ ≤ 30 ns in all cases. (*b*) By scanning the delay *t*_s_ between the signal provided by the PETRA III bunchclock and the sampling instant of the ADC, the QAPD can be used for equivalent time sampling of the preamplified signal. In this mode, individual data points correspond to an average over *N* ≃ 5.2 × 10^5^ iterations (accumulation time 0.1 s per point at *f*_b_ ≃ 5.2 MHz). The optimal sampling instant *t*_s_ is given by the maximum pulse height. It was found that, contrary to the initial expectations, the pulse width does not depend on the number of simultaneously detected photons. Measurements have been performed at a photon energy of *E* = 18 keV during the PETRA III 40-bunch mode.

An exemplary analog signal recorded after several iterative optimization cycles is depicted in Fig. 3(*a*) ▶ for a primary X-ray intensity of *I*_0_ ≃ 6 × 10^7^ photons s^−1^ (detected X-ray photons). The signal rise time (minimum/maximum) of τ ≤ 8 ns is roughly independent of the respective pulse height; the integral pulse length of τ ≤ 30 ns allows for experiments at *f*_b_ ≤ 31 MHz. The average pulse height corresponding to the detection of an individual 18 keV X-ray photon is 8 mV; this signal can clearly be distinguished from electronic noise. At very high intensities a complete re-biasing of the APD within ≤ 30ns is not possible; an offset voltage of ≤10 mV up to the maximum range of the OPA remains. This offset is subtracted in the subsequent digital signal chain as detailed below.

**Figure 4:**
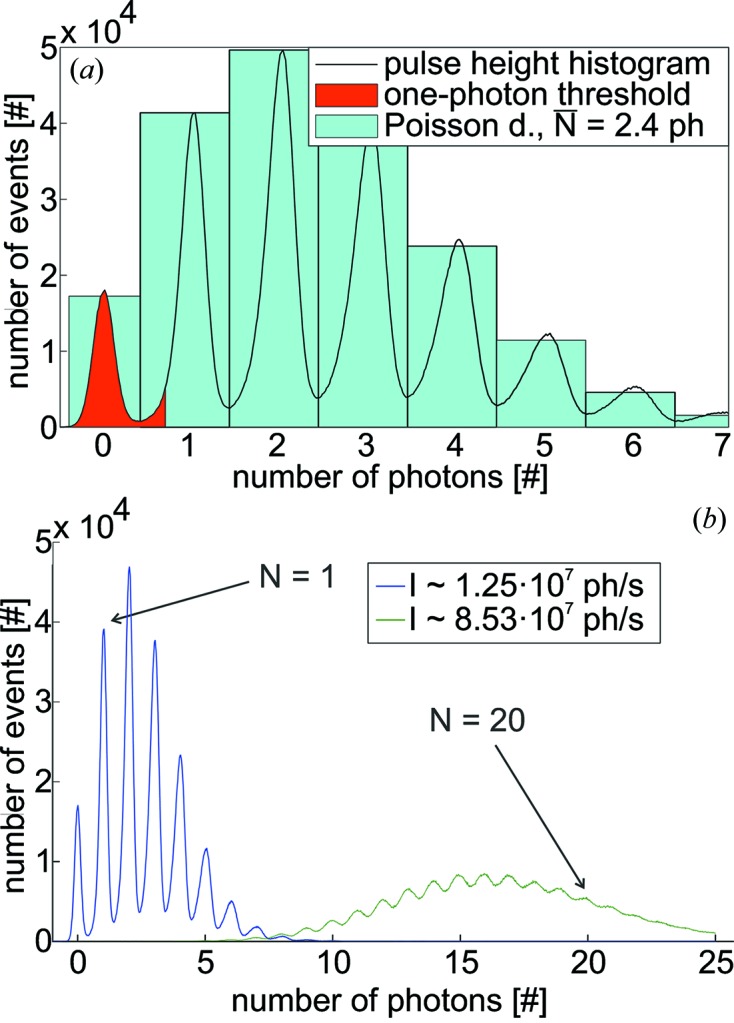
(*a*) A conversion from ADC units to exact photon numbers is made by binning the ADC units according to the distribution observed in a pulse-height histogram. Dead counts are effectively suppressed to ≤0.1 photon s^−1^ by adjusting a user-selectable threshold; the solid red region of the pulse-height histogram is therefore set to zero during the conversion process. The Poisson character of the acquired histograms is verified by an exemplary Poisson fit, plotted in light blue. An average number of 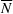 = 2.4 photons pulse^−1^ is determined. (*b*) Pulse-height histograms obtained for two different X-ray intensities. Individual photon numbers can clearly be distinguished up to *N* = 20 photons pulse^−1^; the integral of the respective histograms is proportional to the incident X-ray flux up to *N* ≃ 120 photons pulse^−1^ (limited by the full-scale deflection of the ADC).

The multi-photon counting capability of the preamplifier is demonstrated by means of a pulse-height histogram, plotted in brown in Fig. 3(*a*) ▶. The peak pulse height of each displayed event, at the position marked by a vertical brown dash-dotted line, is therefore recorded. The corresponding histograms exhibit the characteristics of a Poisson distribution [quantified in Fig. 4(*a*) below] which proved that multi-photon events can be distinguished. The main observables during the optimization process were the pulse shape in terms of rise time, possible over- or undershoot, as well as the pulse length, and most importantly the discrimination capability between individual photon numbers in the pulse-height histogram. It was found that the optimal values for the electronic components slightly depend on the X-ray photon energy as well as the bunch frequency (gain *versus* slew rate and bandwidth). A set of parameters satisfying the needs of most experimental situations is given by Reusch (2013 ▶).

The preamplifier is fully DC coupled. Additional features such as micro-controller (µC) based test charge injection and automatic identification are implemented *via* an Atmel ATtiny44 µC on the printed circuit board (PCB) of the preamplifier. A photograph of the fully assembled PCB including electronic connections for signal routing, HV and LV supply voltages and communication with the on-board µC is shown in Fig. 2(*c*) ▶.

### 2.2. High-speed data acquisition at 31 MHz   

The preamplified analog signal is digitized by a four-channel 14-bit high-speed high-bandwidth ADC (sampling rate ≤250 Msps, analog bandwidth 320 MHz; FMC-104, 4DSP, USA). The resulting digital data are directly streamed to a Xilinx Virtex 6 FPGA on an Ml605 FPGA evaluation board. Fig. 2(*a*) ▶ shows a schematic of the timing and data signal pathway.

The ADC has four DC coupled signal inputs. An external clock (*f*_clock_) can be used to synchronize the sampling process to the operating frequency (microwave frequency *f*_acc_ ≃ 500 MHz) of the synchrotron storage ring. The ADC records one sample at each rising edge of the external clock; individual samples are transferred to the FPGA. An additional trigger signal at the bunch frequency *f*_b_ is provided to the FPGA to match the signal processing to the respective filling mode; individual samples are only digitally processed if the trigger signal is high at the sampling instant. In the case of applications at PETRA III, an external clock frequency of *f*_clock_ = *f*_acc_/2 ≃ 250 MHz is chosen [see Reusch *et al.* (2013*b* ▶) for details on the timing scheme of PETRA III]. Clock and trigger signals are provided by the PETRA III bunchclock and adapted to the mode of operation by the FPGA. A precise delay *t*_s_ (resolution ∼78 ps^1^) can be introduced to both signals by the FPGA in order to exactly determine the sampling instant. The exact trigger frequency and FPGA-based digital data processing depend on the mode of operation. Three modes have been implemented: (i) One trigger pulse is generated for each X-ray bunch (each rising edge of *f*_b_); the raw data corresponding to the height of the analog voltage signal are streamed to the PCI port. (ii) Two trigger pulses at *t*_0_ and *t*_0_ + 8 ns are generated for each X-ray bunch. The sample taken at *t*_0_ ns is subtracted from the sample taken at *t*_0_ + 8 ns in order to remove any offset voltage. A delay of 8 ns has been chosen to comply with the rise time (minimum/maximum) *t*_r_ ≤ 8 ns of the analog signal [see Fig. 2(*a*) ▶]. (iii) A number of six-trigger pulses at *t*_0_ and *t*_0_ + (*n*×4) ns (for 1 ≤ *n* ≤ 5) is generated for each X-ray bunch. Samples recorded at *t* ≥ *t*_0_ are summed to increase the signal-to-noise ratio; the sample taken at *t*_0_ is multiplied by five and subtracted from the result to remove any offset voltage. This mode will be referred to as digital integration. Note that in any mode the spacing Δ*t* between subsequent trigger pulses is restricted to integral clock cycles Δ*t* = *n* × (1/*f*_clock_) ≃ (*n*×4) ns.

In summary, four 14-bit digital signals, corresponding to four quadrants on the APD, are transiently recorded by the Virtex 6 FPGA at a sampling rate of up to 31 MHz given by the bunch frequency *f*_b_ of the synchrotron source. This corresponds to a data rate of 4 channels × 2 bytes × 31 × 10^6^ Hz × (14/16) = 217 Mb s^−1^.^2^ As the maximum data rate of widespread external interfaces (*e.g.* USB or 1 GigE) does not allow for a real-time streaming to mass storage devices at these data rates, data are directly transferred to the CPU *via* a PCI-e connection (PCI-e FPGA implementation using Xillybus; Xillybus Ltd, Haifa, Israel). The CPU then decodes the 14-bit signals to 16-bit data streams; the full data stream can be either stored in a fast solid state disk (SSD) or a RAID0 compound of several common magnetic hard disks, the only limit being the capacity of the mass storage devices.

### 2.3. GPU-based online data analysis at 31 MHz   

Depending on the experimental application of the QAPD, it is usually desirable to perform an online data analysis of the acquired waveforms in order to limit the amount of data and facilitate further data processing. Apart from the first basic data reduction performed in the FPGA (*i.e.* offset subtraction, integration), advanced routines are performed by a medium-scale GPU using the Nvidia CUDA architecture. The implementation of GPU-based data processing is relatively fast and easy when compared with FPGA-based approaches; a broad range of online analysis tools meeting the demands of current and future applications can therefore be implemented. Basic examples are, for example, a temporal binning of individual data points (setting a ‘macroscopic’ exposure time) or a position and/or intensity correlation analysis (*i.e.* ‘X-ray photon correlation spectroscopy’). In the context of time-resolved X-ray diffraction, a temporal sorting of samples relative to the stroboscopic experiment is necessary (see Fig. 2*b* ▶). Individual samples are in this case added to registers depending on their relative delay with respect to the excitation. The registers are then read out after a user-selectable exposure time. This results in an averaged pump–probe waveform, tightly sampled at intervals of Δ*t*_2_ = 1/*f*_b_. For the exemplary case of time-resolved experiments in the PETRA III 240-bunch mode, the pump–probe waveform [*e.g.* an intensity trace *II*(*n*,*t*) as in Reusch *et al.* (2013*a* ▶,*b* ▶)] is therefore sampled at a temporal resolution of Δ*t* ≃ 30 ns on the full achievable temporal scale of 0 ≤ *t* ≤ 1 ms (limited by the frequency of typically *f*_pp_ = 1 kHz of the stroboscopic experiment). Finer sampling is achieved by combining multiple experiments for temporally slightly shifted excitations; additional clock I/Os of the Virtex 6 FPGA or an external delay generator can be used for this purpose. A trigger signal at *f*_pp_ is provided to the FPGA to fix the sampling instant of the first sample relative to the excitation.

## 3. Benchmark experiments   

A fully functional prototype of the QAPD including the analog and digital signal chain has been tested at beamline P10 during the 40-bunch mode of PETRA III (*f*_b_ ≃ 5.2 MHz), at a photon energy of 13.8 keV. Experiments have been performed at the Göttingen Instrument for Nano Imaging with X-rays (GINIX); a detailed description of the set-up can, for example, be found by Olendrowitz *et al.* (2012 ▶). In brief, the X-ray beam is focused to ≤500 nm (both directions) by a Kirkpatrick–Baez (KB) mirror pair; slit systems in front of the KB optics allow for a controlled numerical aperture of the focusing optics and the adjustment of the coherence properties. The flux is controlled and varied by a set of attenuators positioned in the unfocused beam. The APD is placed on the optical axis at a distance of ∼50 cm from the focal plane. The beam divergence of ∼1.5 mrad leads to an X-ray spot size of ∼1.5 mm diameter at the surface of the APD.

### 3.1. Timing of the analog-to-digital conversion process   

In the first step of any experiment the exact timing between the analog-to-digital conversion process and the arrival time of the individual synchrotron pulses has to be adjusted. The delay *t*_s_ is therefore scanned *via* the measurement control software *SPEC* (Certified Scientific Software; http://www.certif.com/); the sum of the resulting raw data points for each *t*_s_ (no integration, no offset subtraction) is plotted *versus* *t*_s_ in Fig. 3(*b*) ▶. The scans correspond to the signal of a preamplified pulse averaged over *N* ≃ 5.2 × 10^5^ iterations (accumulation time 0.1 s at *f*_b_ ≃ 5.2 MHz). The detector operates in ‘equivalent time sampling data acquisition’ mode. Measurements of the pulse shape have been recorded for varying primary X-ray intensities in order to check for possible variations (*i.e.* a broadening) of the pulses with increasing X-ray intensity; see Fig. 3(*b*) ▶ for two exemplary measurements at *I* = 6 × 10^7^ photons s^−1^ and *I* = 5 × 10^8^ photons s^−1^. It is found that the averaged pulse shape does not depend on the intensity of the X-ray beam. For succeeding measurements, *t*_s_ is adjusted to match the time point of maximum pulse height.

### 3.2. Poisson character of pulse-height histograms   

The intrinsic linearity as well as the multi-photon counting capability are demonstrated by means of a histogram of the recorded samples [see Figs. 4(*a*) and 4(*b*) ▶]. The sampling instant is in this case set to the maximum in Fig. 3(*b*) ▶. Histograms have been recorded for varying X-ray intensities at an exposure time of 1 s (corresponding to ∼5.2 × 10^6^ samples) each. The Poisson characteristics of the histogram depicted in Fig. 4(*a*) ▶ clearly demonstrate the multi-photon counting capability of the QAPD. The positions of the minima in Fig. 4(*a*) ▶ are used to convert ADC units to photon numbers *via* basic linear binning.

Care has to be taken to suppress any dark counts by introducing a user-selectable threshold, represented by the red shaded area in Fig. 4(*a*) ▶. Note that this procedure induces a non-linearity between the one- and multi-photon events because the bin size for the one-photon event is ∼5% smaller and the number of ‘cropped’ [the red area of the one photon event in Fig. 4(*a*) ▶] photons depends on the intensity of the X-ray pulses. This error will be corrected for by a later version of the GPU-based online data analysis by scaling each one-photon event by a factor corresponding to the ratio of ‘cropped’ photons as determined from future experiments.

The multi-photon counting capability is further quantified by fitting a Poisson distribution to the histograms by means of non-linear least-squares fitting (see Fig. 4*a* ▶). An average number of 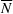 = 2.4 photons pulse^−1^, corresponding to an X-ray flux of *I* = 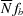 = 1.25 × 10^7^ photons s^−1^ (detected photons) is determined. Individual photon numbers can clearly be distinguished up to *N* = 20  photons pulse^−1^ as demonstrated in Fig. 4(*b*) ▶ for an increased flux of *I* = 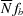 = 8.53 × 10^7^ photons s^−1^. The full-scale deflection of the ADC (±1 V at 14-bits) limits the maximum number of photons to ≤120 photons pulse^−1^.

### 3.3. High dynamic range demonstrated by knife-edge measurements   

**Figure 5:**
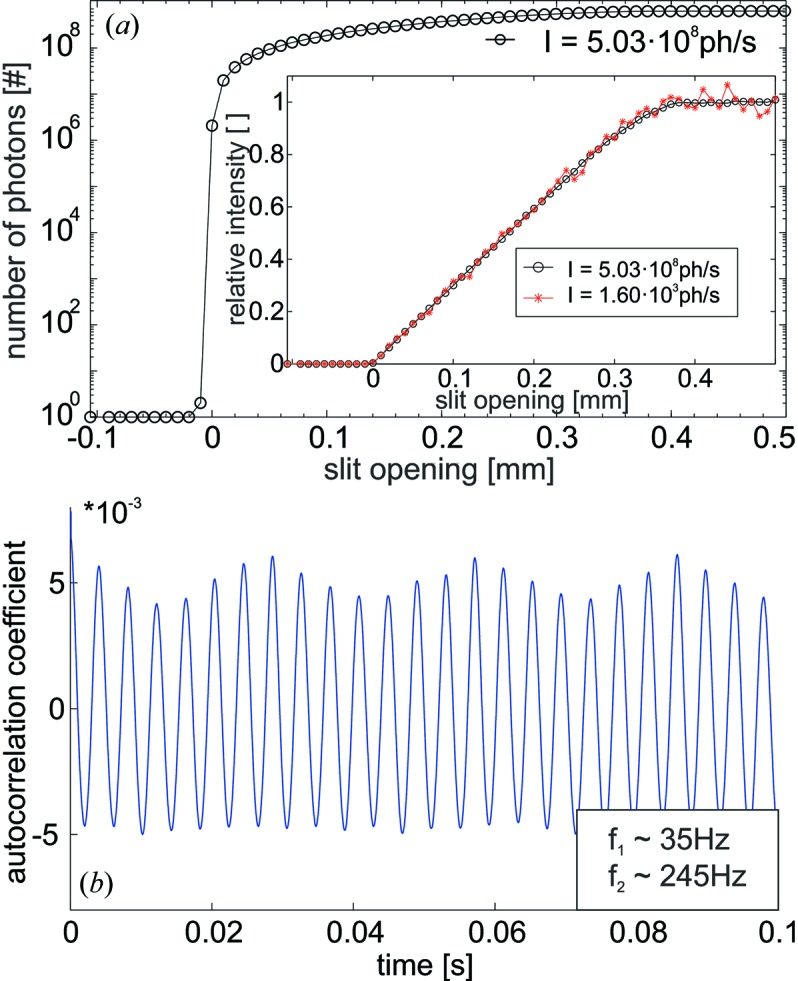
(*a*) Integrated number of photons as a function of knife-edge position (slit system) at a photon energy of *E* = 13.6 keV (40-bunch mode) on a semi-logarithmic scale. Two different primary X-ray intensities (*I* ≃ 5 × 10^8^ photons s^−1^ and *I* ≃ 1.6 × 10^3^ photons s^−1^) have been applied for the measurements presented in the inset; the intrinsic linearity of the QAPD manifests is validated from the perfect overlay of the resulting scans. (*b*) Autocorrelation coefficient of the pulse-wise sampled intensity waveform *I*(*t*). The peak at τ = 0 is omitted. Two characteristic and prominent oscillations at *f*_1_ ≃ 35Hz and *f*_2_ ≃ 245 Hz are observed and can be attributed to mechanical vibrations of the monochromator crystals induced by the cryogenic pumping.

The essentially noise-free single-photon-counting capability in combination with the intrinsic linearity and compatibility with high-flux measurements is demonstrated by a knife-edge scan in front of the KB mirrors (see Fig. 5*a* ▶). As the resulting curves may be influenced by beam inhomogeneities, measurements have been repeated for varying X-ray intensities as depicted in the inset of Fig. 5(*a* ▶). Maximal intensities of *I* = 5.03 × 10^8^ photons s^−1^ and *I* = 1.60 × 10^3^ photons s^−1^ are obtained for attenuator values of *I* = *I*_0_ × 4.43 × 10^−3^ and *I* = *I*_0_ × 1.426 × 10^−8^. This corresponds to a simultaneous detection of *N* = 5.03 × 10^8^ photons s^−1^/5.2 × 10^6^ Hz ≃ 97 photons in the case of the high-flux measurement, not yet limited by the full-scale deflection of the ADC. A perfect overlap of the scans is a proof of the intrinsic linearity of the QAPD and a verification of the multi-photon counting detection scheme. Taking into account the respective attenuator values, primary X-ray intensities of *I*_0_ = 1.13 × 10^11^ ± 5.06 × 10^6^ photons s^−1^ and *I*_0_ = 1.13 × 10^11^ ± 2.80 × 10^9^ photons s^−1^ are obtained for both cases, well in the error intervals given by *I* ^1/2^. In conclusion, depending on the filling mode an X-ray flux of ≥6 × 5.03 × 10^8^ photons s^−1^ ≃ 3 × 10^9^ photons (pixel s)^−1^ can be detected by the QAPD while still resolving individual photons in the low-intensity regions. Note that the presented knife-edge measurements have been performed at *f*_b_ ≃ 5.2 MHz whereas experiments at *f*_b_ ≃ 6 × 5.2 MHz ≃ 31 MHz have been demonstrated in Fig. 5(*a*) ▶.

### 3.4. Auto-covariance analysis reveals characteristic intensity variations of direct X-ray beam   

The temporal resolution of the QAPD has been exploited for pulse-resolved beam characterization experiments at beamline P10 of PETRA III. The beam was centered on one quadrant of the QAPD. An intensity waveform *I*(*t*) of duration 1 s was measured at an average X-ray flux of ∼1.25 × 10^7^ photons s^−1^ [corresponding to the histogram depicted in Fig. 4(*a*) ▶]. The *f*_b_ ≃ 5.2 MHz bunch frequency leads to equally spaced sampling points at integral multiples of *t* ≃ 1/(5.2 × 10^6^) s ≃ 192 ns. The resulting *I*(*t*) was analyzed in terms of the autocovariance,
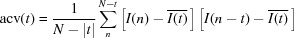
(see Fig. 5*b* ▶), as well as the power spectral density,
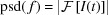
(not depicted here). For Poisson processes, the variance var[*I*(*t*)] is equal to the mean 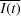 of the intensity signal. The value of the autocovariance acv(*t*=0) at *t* = 0 is in general equivalent to the variance var[*I*(*t*)]. The autocorrelation coefficient acv(*t*)/acv(*t*=0) is therefore in this case a measure of the strength of the intensity variations relative to the mean 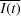.

The autocorrelation coefficient of *I*(*t*) is depicted in Fig. 5(*b*) ▶; the central maximum at *t* = 0 has been omitted. As the width of the maximum is less than *t* = 192 ns, subsequent X-ray bunches are temporally uncorrelated, apart from two characteristic intensity modulations at relative amplitudes of ≤5 × 10^−3^ and frequencies of *f*_1_ ≃ 35 Hz and *f*_2_ ≃ 245 Hz. These modulations can clearly be attributed to mechanical vibrations of the X-ray monochromator.^3^ Let us briefly point out here that, in principle, all characteristic frequencies up to *f* ≤ (1/2) × 31 MHz (the Nyquist limit) can be measured by the pulse-wise detection scheme.

## 4. Summary and outlook: current and future applications of pulse-resolved detection schemes   

We have demonstrated an approach to continuously measure the intensity of individual X-ray pulses at unprecedented repetition rates of up to 31 MHz. The detection and read-out scheme has been commissioned and validated during various experiments at beamlines P08 and P10 at PETRA III. X-ray photon energies of 13.6 keV to 24 keV have been used. A simultaneous detection of *N* ≥ 97 photons per pulse in combination with a clear separation of individual photons from electronic noise in the low-intensity regions leads to a continuous wave dynamic range of ≥5.2 × 10^6^ Hz × 97 photons pixel^−1^ ≃ 5.03 × 10^8^ photons (pixel s)^−1^ in the case of the PETRA III 40-bunch mode, and ≥31 × 10^6^ Hz × 97 photons pixel^−1^ ≃ 3 × 10^9^ photons (pixel s)^−1^ in the case of the PETRA III 240-bunch mode. Most importantly, each event is time stamped, allowing for a pulse-to-pulse characterization of fast dynamics in the context of time-resolved X-ray diffraction experiments. Online data analysis at a rate of ≥31 MHz is performed by a medium-scale GPU. We also want to stress that, while the current detector performance characterization has been performed at high monochromaticity [Si(111) double-crystal monochromator], a significantly broader spectral bandwidth would also be tolerable. If multi-photon detection of maximally *N* photons per pulse is required, the allowable spectral bandwidth is roughly (Δ*E*/*E*) = 1/*N*, in order not to compromise proper counting of photons. Therefore one has to judge the allowable spectral bandwidth with respect to the maximum signal for each experiment separately. We stress that the intrinsic bandwidth of pink undulator radiation at PETRA III would still be well compatible with counting of 100 photons pulse^−1^.

The high dynamic range of the QAPD relaxes the constraints of using X-ray attenuators, simultaneously increasing the achievable signal-to-noise ratio. A temporal binning of the data points is implemented in the GPU, allowing basically arbitrary short exposure times down to a single X-ray pulse to be set.

Our primary goal is, however, to use the QAPD in order to measure fast dynamics in the pump–probe diffraction scheme as well as in coherent imaging, both for equilibrium and driven soft matter and biomolecular systems. As illustrated above, the operating principle of the QAPD allows in these cases for a tremendous reduction in measurement time, extending the range of possible sample systems, diffraction experiments and environmental conditions. Furthermore, experimental constraints for time-resolved experiments are greatly relaxed. Contrary to experiments using high-speed choppers or gated pixel detectors, which are limited to dedicated ‘low frequency’ filling modes, the presented detection scheme is applicable in a much broader parameter range. A major disadvantage/limitation of the system is the fact that it currently has only 1–4 pixels, ruling out the use as the main detector in many experiments necessitating one- or two-dimensional readout. On the other hand, the QAPD is highly portable and easy to implement into any beamline layout, for example as a pulse-to-pulse diagnostic tool or as a point detector for anisotropic diffraction signals (Bragg peaks, reflectivity). Time-resolved experiments are therefore no longer limited to specialized endstations, suitable reference timing signals provided.

In addition to the significant benefit for pump–probe experiments, in particular in the case of irreversible phenomena, pulse-by pulse readout can enable pulse-resolved beam monitoring, and extend the capabilities of high-speed X-ray photon correlation spectroscopy and X-ray imaging experiments, taking advantage of the significantly enhanced dynamic range and the demonstrated MHz readout rate. For studies of interface dynamics, where the specular and off-specular reflectivity or signal can be very suitably probed by point detectors, an extended time window and increased dynamic range over current APD detection technology can be anticipated. This can serve, for example, studies of undulation modes in oriented lamellar phases of smectic liquid crystalline symmetry (Constantin *et al.*, 2006 ▶), or multilamellar lipid membranes, closing the gap between XPCS and neutron spin echo experiments (Rheinstädter, 2006 ▶). For the case of phase-contrast imaging of periodic dynamics, a time scan of each pixel in the raster scan of the sample can be performed with the help of the QAPD in differential phase-contrast mode, circumventing the current limitations’ temporal range, data accumulation time and/or bunch mode constraints. Very significant and important examples in this time-resolved imaging mode have already been reported for driven magnetic dynamics (Stoll *et al.*, 2004 ▶).

When compared with concurring approaches [*e.g.* the AGIPD (Henrich *et al.*, 2011 ▶), the CSPAD (Herrmann *et al.*, 2013 ▶), the Analog integrating pixel array detector (Koerner & Gruner, 2011 ▶) or the single-photon-counting XNAP (Fajardo *et al.*, 2013 ▶)], the digitalization of an unlimited number of subsequent X-ray pulses at a temporal spacing of ≤30 ns, the easy integration into any beamline set-up at synchrotron or FEL sources (suitable timing signals provided), the high flexibility of the quadrant approach and the comparatively low production cost can make the QAPD a useful device. Future improvements include the implementation of fully differential analog signalling as well as extensive testing of alternative detector front-ends (including CVD diamond, GaAs and CdTe-based materials).

## Footnotes

1
http://www.xilinx.com/support/documentation/data_sheets/ds152.pdf.

2 One 16-bit integer is encoded by 2 bytes.

3
http://hasyweb.desy.de/science/annual_reports/2007_report/part1/contrib/28/22252.pdf.
